# Self-Excited Acoustical Measurement System for Rock Mass Stress Mapping

**DOI:** 10.3390/s21206749

**Published:** 2021-10-11

**Authors:** Krzysztof Lalik, Ireneusz Dominik, Krzysztof Skrzypkowski, Waldemar Korzeniowski, Krzysztof Zagórski

**Affiliations:** 1 Faculty of Mechanical Engineering and Robotics, AGH University of Science and Technology, Al. Mickiewicza 30, 30-059 Kraków, Poland; dominik@agh.edu.pl (I.D.); zagkrzys@agh.edu.pl (K.Z.); 2 Faculty of Civil Engineering and Resource Management, AGH University of Science and Technology, Al. Mickiewicza 30, 30-059 Kraków, Poland; skrzypko@agh.edu.pl (K.S.); walkor@agh.edu.pl (W.K.)

**Keywords:** ultrasonic systems, vibrodiagnostics, smart sensors, bolting, stress measurement

## Abstract

This paper presents the results of a preliminary study of a self-excited acoustical system (SAS) for nondestructive testing (NDT). The SAS system was used for mine excavation stresses examination. The principle of operation of the SAS system based on the elastoacoustical effect is presented. A numerical analysis of the excavation was carried out considering the stress factor. An equivalent model based on a two-degree-of-freedom system with a delay has been developed. This model allowed to determine the relation which relates the frequency of the self-excited system to the stress level in the studied ceiling section. This relationship is defined by the elastoacoustic coefficient. The test details for anchorages in laboratory conditions and Wieliczka Salt Mine were presented. This research details of a method for creating actual stress maps in the ceiling of a mine excavation. The results confirmed the possibility of using the new measurement system to monitor the state of stresses in the rock mass.

## 1. Introduction

In mining, one of the most important aspects is to ensure human safety in changing environmental conditions [1,2]. The risk may result from both mining operations [3,4], as well as movements of the rock mass itself [5,6]. Although there are methods of protection such as anchor bolt [7] it is still essential to know the state of stress in the mine excavation in order to prevent death and severe injury to miners. For coal mining, stress monitoring systems have been shown in [8]. The authors present the primary indicators for the quantitative assessment of stresses in the excavation. The classical methods of measuring the roof displacement are used. An indicator for the strength assessment of anchors is also given. The following parameters are measured: arch strength, geostress, lateral pressure coefficient, and surrounding rock mechanical parameter. However, all of these methods require destructive testing in rock drilling or mechanical tearing of the anchors. An analytical approach to stress propagation using the method of tension is presented in [9]. The results were compared with experimental destructive test results in the existing rock mass. Test results for a combination of numerical simulations and destructive experimental tests were presented for investigating the failure behavior of rock massif in [10]. An interesting solution is the measurement of acoustic emission used in [11]. The authors show the correlation between the stress state of the specimens and the change in the acoustic emission coefficient for coal. In ore mining, methods related to the measurement of the anchorage itself are widely used [7]. It is due to the different dynamics of rock mass stress changes than in coal mining [12]. In the vast majority of cases, the change is of an impact nature. In salt mining, another fascinating measurement aspect has been claimed in the form of fatigue testing. The authors in [13] present laboratory fatigue test results for salt from a salt chamber that is used as compressed air storage for wind energy conversion. The salt chamber is used as an air tank. The self-healing capacity of salt achieves air-tightness due to viscoplastic deformation of the grains. New measurement methods and new sensors are constantly being explored to improve the miners’ safety. The new methods based on wireless sensors are presented in [14,15]. The hydraulic fracturing test was conducted, and the results were presented in [16,17,18]. The test proved it was difficult to make artificial fractures because of water leaking between the rock layer. The primary purpose of [19] was to establish the relationship between measured in situ stress data by a neural network. The electromagnetic radiation of rock to perform the internal stress distributions was done by [20]. The self-excited acoustic SAS system is an indirect structural stress measurement system based on the elastoacoustic effect [21]. It has been successfully applied to measure stresses in metal [22] and concrete [23]. Attempts have also been made to implement it for single anchors [24]. Artificial intelligence was also used in the articles [25]. The fuzzy logic was used for the interpretation of the results. In each case, however, the system was attached to materials made of a single material. This paper presents an attempt to implement it as a sensor placed on two composite anchors to determine the stresses in the rock mass, which is located between these two anchors. A numerical analysis of the anchored rock mass model was carried out, and a 2-DOF model of the measuring system was made. The presented measuring system is a key element in monitoring the stresses state in the excavation and thus contributes to improving the safety of people in the salt mine.

## 2. Methodology

**Figure 1:**
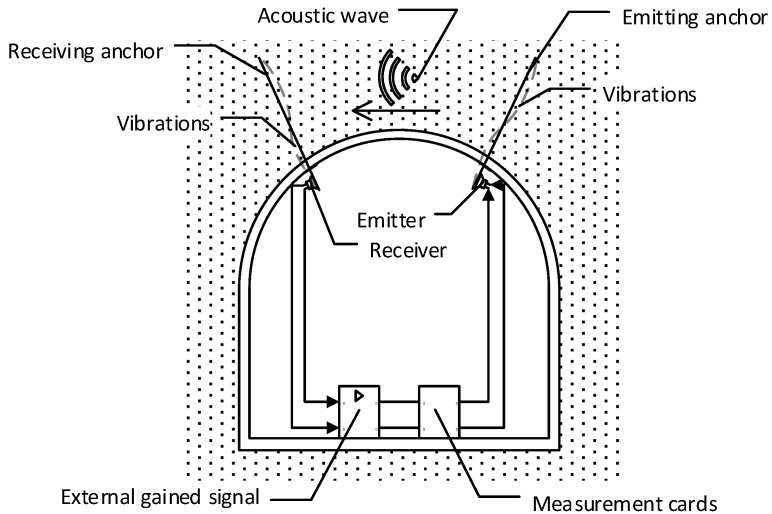
Example of application of SAS system in a drilled gallery with circular vault-system with separated transmitting and receiving head.

The conceptual scheme of the SAS system application is to monitor the stress state of the roof over the entire mining roadway and create a dynamic stress map of the excavation is shown in Figure 1. In this case, the transmitting head is the entire anchor. The receiving antenna can be attached to any other anchor. The measurement transducer works in a heterodyne system. The receiver and transmitter of the acoustic wave are located at a certain distance. In an autodyne system, both heads are located in the same place. The exciter and receiver axes are perpendicular to the axes of the anchors to which they are attached. It ensures the best transmission of the acoustic wave through the rock mass.

The proposed system works on a principle very similar to a radio network. The transmitting antenna, i.e., the anchor on which the inductor is placed, vibrates and emits acoustic waves. The waves flowing through the roof reach the receiving antenna, i.e., the anchor on which the receiver is placed. In this case, the receiver and inductor create a closed circuit with positive feedback. The signal from the receiver is amplified appropriately using an external power source and then routed back to the inductor. The resulting induced waves have a specific frequency that depends on the strain of the rock mass and the type of rock between the two anchors. Open systems, i.e., those in which modal frequencies of free vibrations are measured, have fundamental problems related to wave phenomena occurring at the media boundary. There are simple problems with the interpretation of the obtained frequency spectra. In the case of a closed, positive feedback loop, the amplification of a particular frequency of the limit cycle of the self-excited system is obtained. No limit cycle phenomenon appears in open systems. Therefore, it is necessary to loop the entire system.

**Figure 2:**
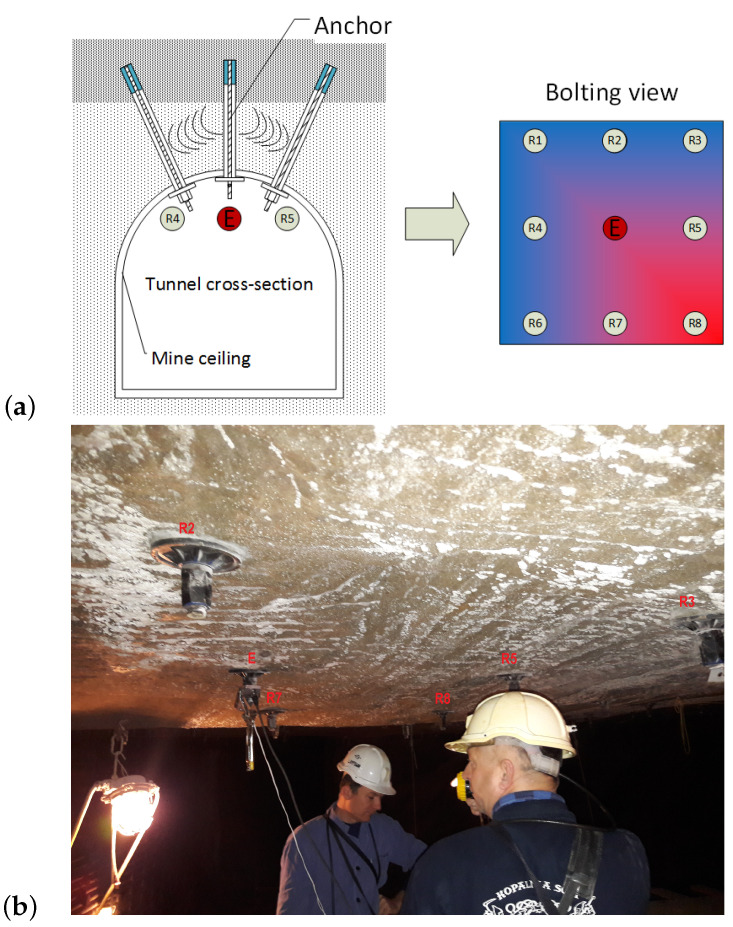
Schematic of a system with multiple receivers on multiple anchors: (**a**) Diagram, (**b**) Real mine view with labelled anchors.

The proposed system may consist of a single transmitting anchor, multiple receiving anchors, and an appropriate switching system. A schematic of such a system is shown in Figure 2a. By causing cyclic switching of the receiving antennas, a measurement system can be created that continuously monitors the excavation condition over a large area. In Figure 2a, the blue color indicates the area where the initial conditions have not changed. The red color indicates that there has been a change in the heterodyne frequency in the inductor E-receiver R8 system due to a change in stress in the direction determined by the straight line passing between the heads. Figure 2b shows the reference position of heads R1-R8 and emitter E showed in Figure 2a on the actual mine roof.

**Figure 3:**
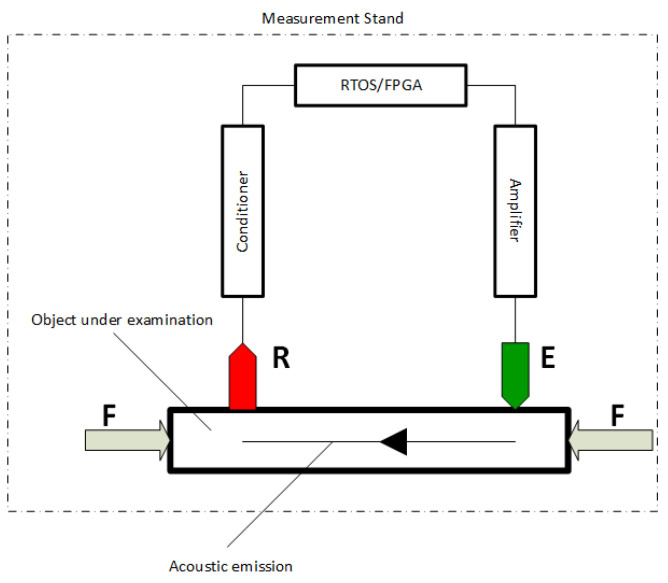
Schematic of the Self-Excited Acoustical System.

A schematic of the SAS system, which is an example of an auto-oscillator, is shown in Figure 3. The system can be broadly divided into two parts. The first part is the test object. The second part of the system is the actuator part, which has two main components: the exciter (E)—a piezoelectric actuator and the receiver (R)—a piezoelectric accelerometer sensor. The system uses an IMI 623C01 piezoelectric accelerometer with a VibAMP PA-3000 conditioner. The parameters of the accelerometer are given in Table 1, and the actuator in Table 2. The amplifier, actuator (E), conditioner, and accelerometric sensor (R) realize positive feedback. The accelerometer signal is conditioned to a voltage signal by a conditioner. The measurement system is implemented by the Field Programmable Gate Array (FPGA) system. The FPGA has two functions. The most important is to pass the signal to the amplifier directly, from where it goes to the exciter (E). It creates the feedback loop. The second function is to prepare a buffer of measurement data for the Real-Time Operating System (RTOS), which can then be processed or archived. By using the electrical loop, non-extinguishable oscillations with a specific limit cycle frequency are generated. The main factor affecting the self-excited system frequency is the change in the propagation velocity of the wave, and hence the change in the time for the wave to pass through the test object. Changes in stress cause changes in the speed of propagation of the acoustic wave. Measuring the frequency of the limit cycle indicates the level of stress in the excavation.

## 3. System Modelling

**Figure 4:**
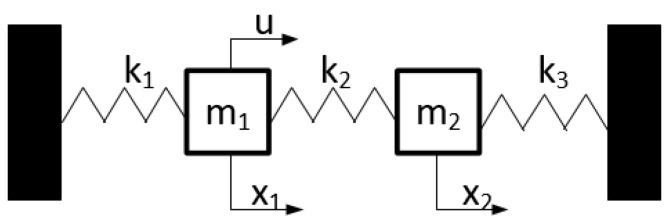
Reduced 2-DOF model of the SAS system.

Two masses represent the dynamic model with spring rates. The scheme shown in Figure 4 corresponds to the scheme of the SAS system. Mass $m_{1}$ represents the transmitting anchor, mass $m_{2}$ represents the receiving anchor. The elasticities $k_{1}$,$k_{2}$,$k_{3}$ represent the elasticities of the rock mass surrounding the anchorage.

The state equations for such an arrangement are determined by Equations (1) and (2). $x_{1}$ and $x_{2}$ are the respective displacements of the transmitting head and the receiving head.

(1) $$m_{1}{\ddot{x}}_{1}+\left(k_{1}+k_{2}\right)x_{1}-k_{2}x_{2}=u\left(t\right)$$

(2) $$m_{2}{\ddot{x}}_{2}+\left(k_{2}+k_{3}\right)x_{2}-k_{2}x_{1}=0$$

The signal from the receiving bolt goes to the transmitting bolt using positive feedback. Hence, the forcing force u(t) can be determined by the control law given by Equation (3):

(3) $$u\left(t\right)=K_{c}\times x_{2}\left(t-\tau_{t}\right)$$

where:

$K_{c}$—Gain,
$\tau_{t}$—Signal delay.

A steady-state solution is given by Equation (4) for mass $m_{1}$ and by Equation (5) for mass $m_{2}$.

(4) $$x_{1}=A_{1}\sin\omega t$$

(5) $$x_{2}=A_{2}\sin\omega t$$

where:

$A_{1}$,$A_{2}$—Amplitudes of vibration for corresponding bolting,
ω—SAS frequency.

After substituting Equations (4) and (5) into Equations (1) and (2) and ordering, the polynomial Equation (6) is obtained from which the system frequencies can be calculated as a function of delay assuming equal system masses $m_{1}=m_{2}=m$.

(6) $$0=\omega^{4}-\frac{k_{1}+2k_{2}+k_{3}-K_{c}\tau_{t}}{m}\omega^{2}-\frac{k_{2}^{2}+K_{c}\tau_{t}\left(k_{2}+k_{3}\right)}{m^{2}}$$

The relation between the stresses and the total delay time in the system is given in [21]. This relationship is expressed by Equation (7).

(7) $$\tau_{t}=\left(\frac{1}{\beta \sigma +1}+\frac{\sigma }{E}\right)t_{0}$$

where:

β—Elastoacoustic coefficient,
σ—Stress,
*E*—Young modulus,
$t_{0}$—Time of acoustic wave propagation in no-stress state.

Relationships (6) and (7) may allow for direct determination of stresses in the investigated rock mass considering the measured frequency of the SAS system. Nevertheless, this method requires further research to identify individual elasticities and masses for different types of roofs and anchors. Such studies will be performed at a later stage under actual mine conditions.

## 4. Stress Modeling in a Mine Roof

**Figure 5:**
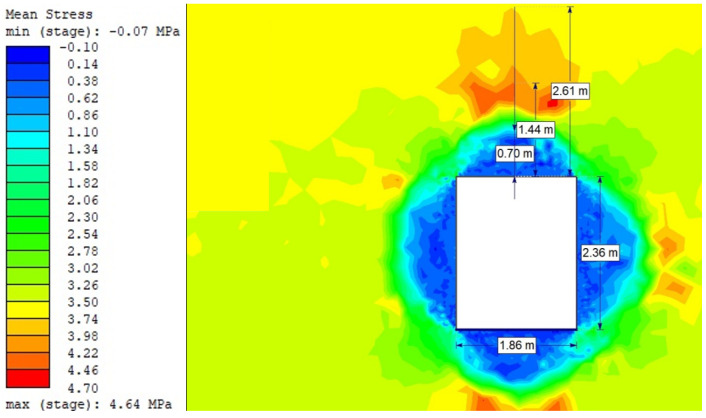
Mean stress for Vernier excavation without bolting (Hoek–Brown criterion).

**Figure 6:**
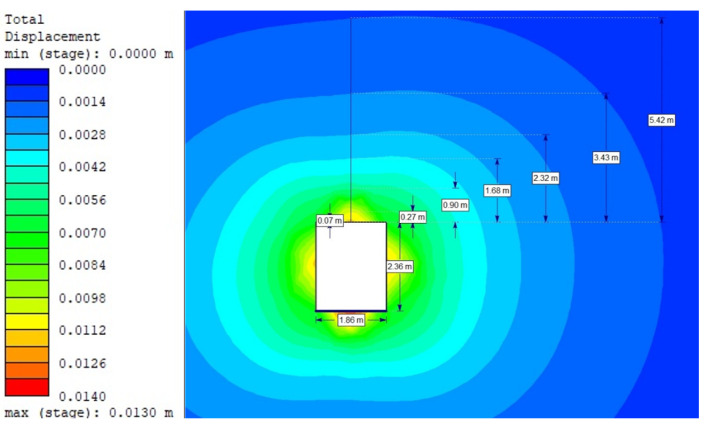
Total strain for Vernier excavation without bolting (Hoek–Brown criterion).

**Figure 7:**
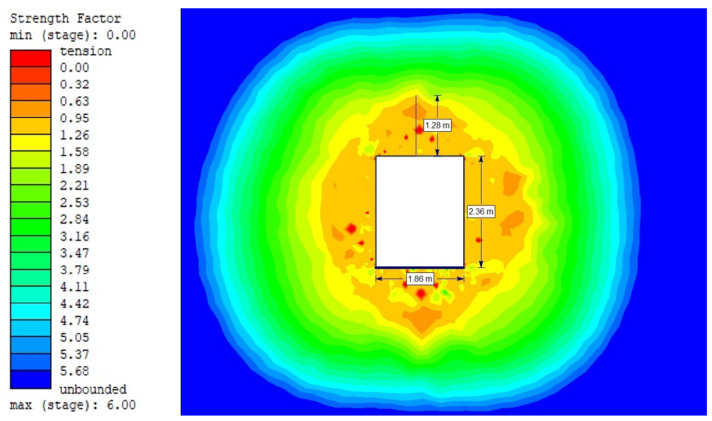
Strength Factor for Vernier excavation beam without bolting (Hoek–Brown criterion).

**Figure 8:**
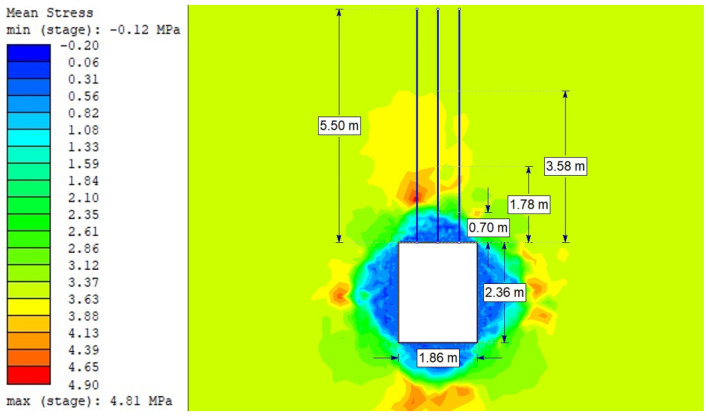
Mean stress for Vernier excavation with full-length anchor bolting (Hoek–Brown criterion).

**Figure 9:**
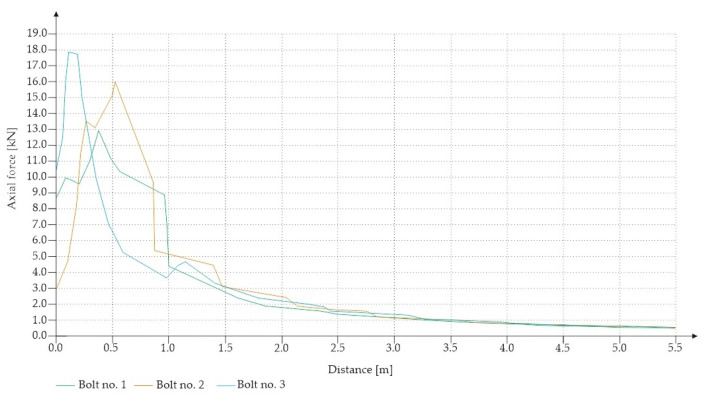
Total strain for Vernier excavation with full-length anchor bolting (Hoek–Brown criterion).

**Figure 10:**
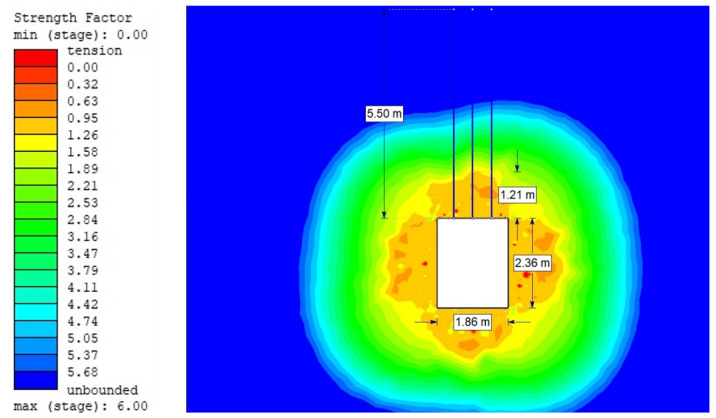
Strength Factor for Vernier excavation beam with full-length anchor bolting (Hoek–Brown criterion).

**Figure 11:**
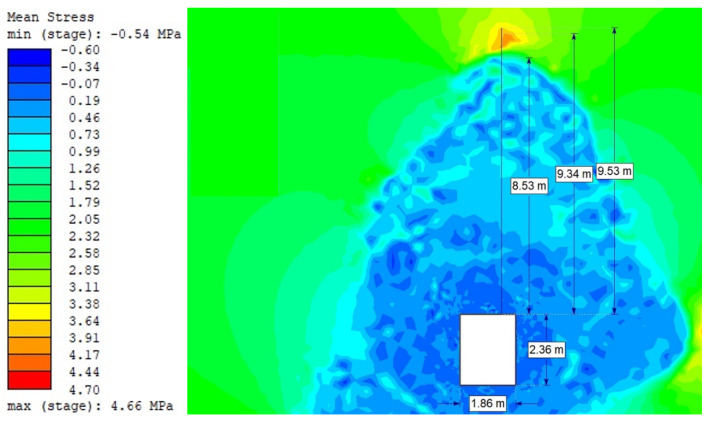
Mean stress for Vernier excavation without bolting (Coulomb–Mohr criterion).

**Figure 12:**
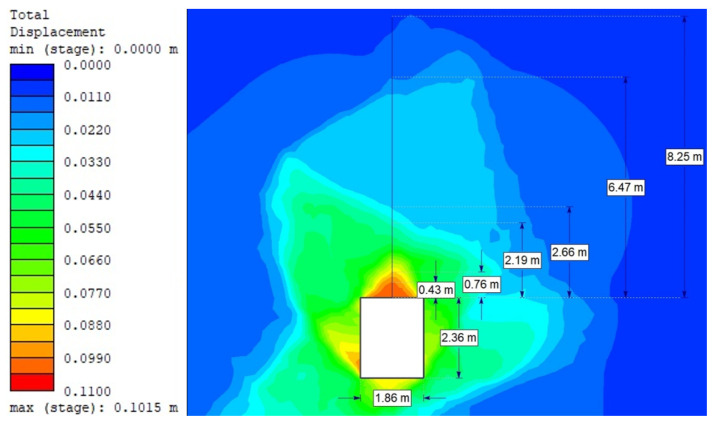
Total strains for Vernier stringers without bolting (Coulomb–Mohr criterion).

The numerical studies were performed in the Phase2D computer program based on the finite element method. The method allows for the approximate solution of physical problems, generally defined by a system of differential equations with appropriate boundary conditions. As a result of solving complex systems of differential equations, we obtain specific function values at selected points. In this method, the considered area is discretized into an equivalent system of a finite number of sub-areas of a simple shape (triangle) called finite elements. This set of elements is connected at points called nodes. Several elements can be connected at each node. A finite element mesh thus replaces the area under consideration. As a result of solving the system of equations at the nodes of the finite element mesh, the values of displacements and forces (reactions) caused by the loads or displacements (deformations) acting on the area are obtained. By having the displacements of the nodal points, the deformations, and then the stresses are calculated. The primary objective of this study was to determine the state of stress, strain, and rock failure zone around the Vernier excavation on level IV at Wieliczka Salt Mine. In addition, the maximum axial force in the excavation anchors was determined for the excavation with shoring. Numerical modeling was performed for the excavations with anchor bolt shoring fixed along their entire length. The strength and elastic parameters of the rock mass were selected based on the geological documentation of Wieliczka Salt Mine, whereas the material constants were calculated using RocLab software. The results of calculations conducted in Phase2D software are presented in the form of Table 3 and Figure 5, Figure 6, Figure 7, Figure 8, Figure 9, Figure 10, Figure 11 and Figure 12 showing, among others: average stress, total strain, range of rock stress in roof and maximum axial force in roof anchors. The Hoek–Brown criterion, Coulomb–Mohr criterion, and Strength Factor strain rate were used in the study. The Hoek–Brown strength condition defines an empirical relationship between standard stresses and the compressive strength of rocks and parameters $m_{b}$, *s* and *a* (characterizing the rock mass quality) selected using Rocklab software or based on tables. The general form of the Hoek–Brown condition, as determined by testing rock samples, is expressed by Equation (8).

(8) $$\sigma_{1}^{{}^{\prime }}=\sigma_{3}^{{}^{\prime }}+\sigma_{ci}^{{}^{\prime }}{\left(m_{b}\frac{\sigma_{3}^{{}^{\prime }}}{\sigma_{ci}^{{}^{\prime }}}+s\right)}^{a}$$

where:

$\sigma_{1}^{{}^{\prime }}$, $\sigma_{3}^{{}^{\prime }}$—Effective maximum and minimum stress at failure,
$\sigma_{ci}^{{}^{\prime }}$—The limit strength of the rock material in uniaxial compression,
$m_{b}$—Value of the Hoek–Brown constant for the rock mass,
*s*, *a*—Empirical constants determined on the basis of rock mass properties tests.

The constants *s* and *a* are determined on the basis of laboratory tests of rock samples in the triaxial state of stress. The constant $m_{b}$ is determined from the empirical relation (9)

(9) $$m_{b}=m_{i}e^{\left(\frac{GSI-100}{28-14D}\right)}$$

where:

- $m_{i}$—constant for unruptured rock, depending on the type of rock, determined using a triaxial compression test or from tabular data,
- GSI—Geological Strength Index,
- *D* —the factor of weakening of the rock mass resulting from the mining method.

In the case when GSI>25, then the parameters *s* and *a* of the Hoek–Brown condition are determined using relations (10) and (11).

(10) $$s=\exp\frac{GSI-100}{9-3D}$$

(11) $$a=\frac{1}{2}+\frac{1}{6}\left(e^{\frac{-GSI}{15}}-e^{\frac{-20}{3}}\right)$$

When GSI<25, the constant Hoek–Brown criterion *s* and *a* are determined from the relationship: s=0; a=0; 65−GSI/200.

The Coulomb–Mohr criterion describes the linear relationship between normal and tangential stresses (or maximum and minimum normal stresses) in the damaged zone. The quantities needed to determine the relationship are defined by Equations (12) and (13)

(12) $$\sigma =\frac{\sigma_{I}+\sigma_{III}}{2}-\frac{\sigma_{I}-\sigma_{III}}{2}\sin\phi$$

(13) $$\tau =\frac{\sigma_{I}-\sigma_{III}}{2}\cos\phi$$

By using expression (14) that defines the stresses σ and τ in the boundary condition equation at the slip surface:

(14) |τ|=σtanϕ+c

Equation (15) was obtained:

(15) $$\frac{\sigma_{I}-\sigma_{III}}{2}\cos\phi =\frac{\sigma_{I}+\sigma_{III}}{2}\tan\phi -\frac{\sigma_{I}-\sigma_{III}}{2}\frac{\sin\phi^{2}}{\cos phi}+c$$

where:

σ,τ—Normal and shear stresses on the slipping surface,
$\sigma_{I}$—Maximum normal stress,
$\sigma_{III}$—Minimum normal stress,
$\frac{\sigma_{I}+\sigma_{III}}{2}$—Horizontal coordinate of the center of Mohr’s circle,
$\frac{\sigma_{I}-\sigma_{III}}{2}$—Radius of Mohr’s circle,
ϕ—Internal friction angle,
*c*—Material cohesion.

The Strength Factor (SF), which expresses the ratio of rock strength to reduced stress at a given point, was used to analyze and determine the failure zones of the rock mass around the workings in the numerical model. If the SF value is less than 1, it means that the reduced stress exceeds the strength of the rock mass at the point, and material failure may occur (plastic analysis). Assuming that the system is elastic, material failure does not occur.

The following parameters were adopted in the numerical modeling: compressive strength Cs=31.14 MPa; tensile strength Ts=1.02 MPa; unit weight γ=0.022 $\mathrm{MN}/m^{3}$; Poisson’s ratio ν=0.44; Young’s modulus E=1410 MPa; Geological strength index GSI=60; friction angle $\phi =46.858^{\circ }$; cohesion c=0.898 MPa; $m_{b}=2.876$; s=0.012; a=0.503; field stress $\sigma_{1}=\sigma_{3}=\Sigma_{Z}=3.74$ MPa.

**Figure 13:**
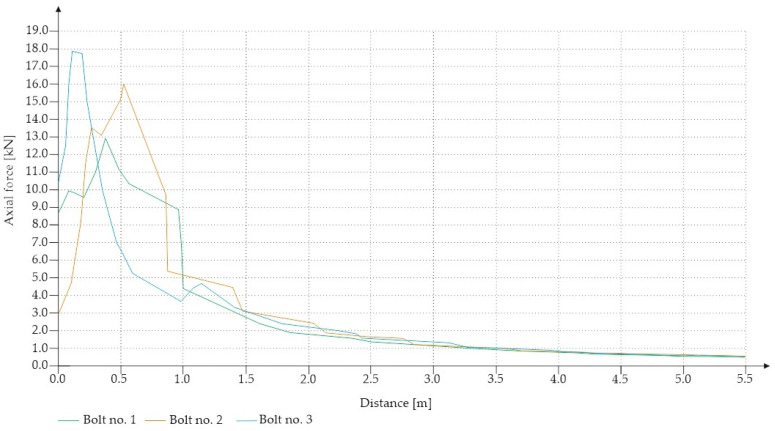
Maximum axial force in full-length anchors for Vernier excavation (Hoek–Brown criterion).

For the Hoek–Brown criterion and unanchored gallery, the results of numerical analysis are presented for the mean stress (Figure 5), total strain (Figure 6), and SF ratio (Figure 7). The white rectangle illustrates the cross-section of the mine roadway. It can be seen that there is a high concentration of stresses above the excavation. For the Hoek–Brown criterion and the sidewalk anchored along its entire length, the results of the numerical analysis are presented for the mean stress (Figure 8), total strain (Figure 9), and SF coefficient (Figure 10). The presence of anchors reduced the stresses and strains above the excavation. Figure 11 shows the distribution of mean stress, and Figure 12 the total strain for the unanchored heading obtained using the Coulomb–Mohr criterion. The strength criterion used shows the same maximum stress values but is located significantly higher above the sidewalk. The proposed Hoek–Brown criterion was also used to calculate the force distribution along the entire length of the modeled anchor. The simulation results are shown in Figure 13.

## 5. Results

**Figure 14:**
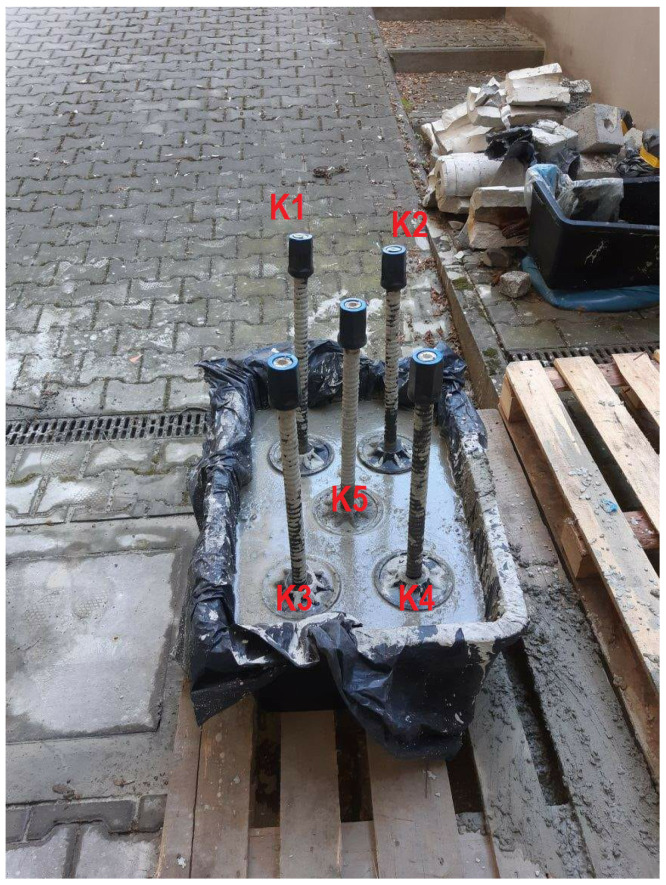
Laboratory model of bolted roof.

The research on stresses in the composite anchor bolt casing type J64-27 was conducted on the laboratory test stand and the Wieliczka Salt Mine. The view of the laboratory model station is shown in Figure 14.

The laboratory measurement methodology consisted of compressing the concrete model with a force of 20 kN at specified locations and directions. The loading force application locations were selected so that there were two anchors in the axis of the applied force. The selected directions of force application were K1–K2, K3–K4, K1–K3, and K2–K4. The SAS system was then attached to specific horizontal locations K1–K2, K3–K4, vertical locations K1–K3, K2–K4, and oblique locations K1–K5, K2–K5, K3–K5, and K4–K5. The highest stress in the tested excavation model had to occur in the direction of the force application. Measurements were performed ten times for each SAS configuration. The comparative results are shown in Table 4. The frequency difference for the direction of the main force application was the largest concerning the second pair of anchors. The table should be interpreted as follows: For the K1–K2 load application direction, the reading from the SAS system setup on the K1–K2 anchors was 51.2 ± 0.1 Hz greater than for the K3–K4 configuration, with the same load configuration (on the K1–K2 direction). The oblique directions K1–K5 and K2–K5 reached a frequency value 16.7 ± 0.3 Hz more than the side direction K3–K4. A similar relationship was seen for every other load configuration.

The preliminary laboratory tests confirmed that it is possible to quantify the stress increase in the tested anchored material using the SAS system. As a general rule, the SAS system indicates higher frequencies for a head application direction consistent with the normal stress direction.

**Figure 15:**
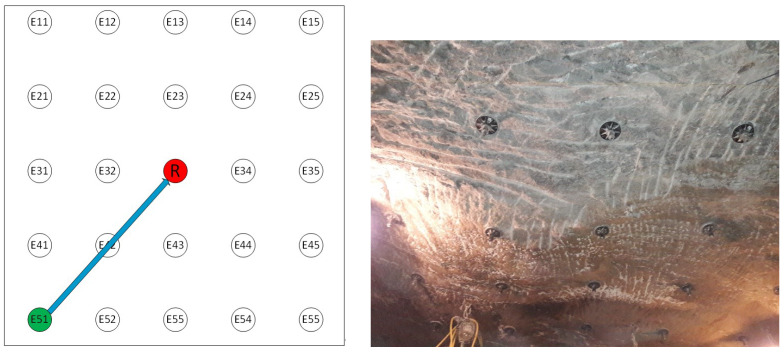
Diagram of anchor head arrangement and view of the actual bolted roof.

In the following part of the task, preliminary investigations were carried out in the transmitting- receiving casing system, which created the first stress maps of the mine excavation. These tests confirmed that an acoustic wave generated at one anchor could be received at another anchor. As part of the research, an experiment was conducted in the mine excavation at Wieliczka Salt Mine. A gallery was prepared in which 25 of J64 anchors with complete bonding were previously anchored. These anchors were arranged in five rows and five columns, whose mutual distance was 1 m (Figure 14). In order to facilitate the experiment description, each anchor was marked with two coordinates: the x-index and the y-index. Figure 15 shows the mounting directions of the receiving head (position 33) and the sample transmitting anchor (E51). The tests were conducted under different load application arrangements. All anchors were not tightened through the nut.

**Figure 16:**
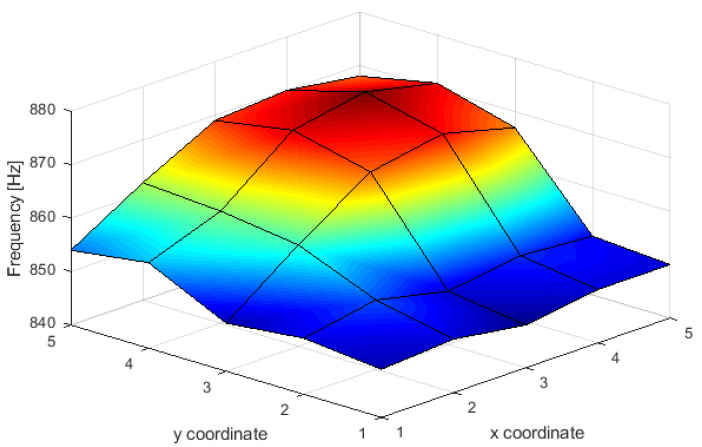
Frequency values in nodes with anchors.

For the tests, individual anchors or anchor assemblies were tightened to a torque of 200 Nm, causing local compression of the excavation. A transmitting head was then applied to each anchor, and the limit cycle frequency for a given head position configuration ($E_{x}y-R$) was read. Each frequency was then entered into a matrix (x,y,f) where x,y are the coordinates of a given anchor, and *f* is the frequency value for a given emitter position. An example of the stress field is shown in Figure 16.

**Figure 17:**
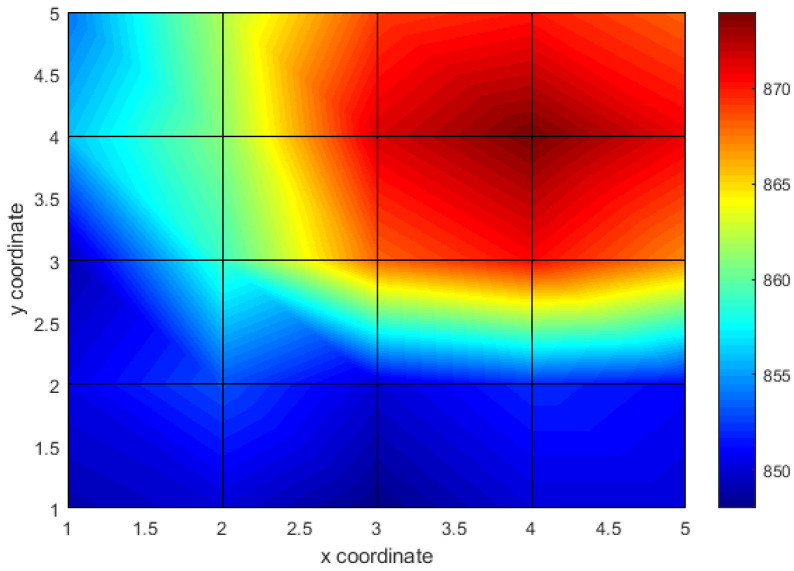
Stress field plan view.

In the position projection, where frequency values are represented by color change, interpretation of the results is even easier (Figure 17). In the presented case, only the anchor with coordinates 4.4 was tightened with the nut. The frequencies were measured only in the nodes. The coordinate intersections and the space between them were interpolated. Nevertheless, it is easy to see that the highest frequency is achieved for the exact coordinates as the compressive stress is applied.

## 6. Conclusions

The paper presents a proposal for the new ultrasonic stress monitoring system for a mine excavation. It also presents results of preliminary tests in a laboratory system and undermining conditions. The measurement system is based on the self-excitation effect. It brings the system to the limit cycle, where the frequency depends on the stresses in the tested space between anchors. The elastoacoustic effect pairs the stress change and the frequency of the limit cycle. This effect determines that as the stress changes, the propagation speed of the acoustic wave in the test material changes. It changes the wave transmission time between the transmitting and receiving antenna, which directly affects the limit cycle frequency.

The proposed 2-DOF model, which is equivalent to the real system, allowed to determine the relation that relates the frequency of the self-excited system to the stress level in the studied ceiling section. This relationship is determined by the elastoacoustic coefficient. This relationship was formed by considering the acceleration form of the feedback signal and the effect of signal delay on the limit cycle frequency of the SAS system.

The laboratory and in situ tests performed allowed to conclude for the SAS system:

- The proposed reduced model can be used in the future to determine the absolute value of stress between anchors based on the measured frequency of the SAS system.
- It is possible to quantify the stress increase in the tested anchored specimen with the SAS system. As a general rule, the SAS system indicates higher frequencies for a head application direction consistent with the predominant stress direction.
- The presented results of preliminary tests at the Wieliczka Salt Mine allowed confirming the applicability of the measurement system in the conditions of a real mine.

The main advantage of the proposed method over the other mentioned methods is the simplicity. On the one hand it is the simplicity and speed of the measurement method itself. Neither roof of the excavation nor anchors have to be specially prepared and do not require long lasting preparations. The limit cycle is reached in less than 1 s and in principle this moment is sufficient to end the measurement itself, although it can also be continued. Such a system can be used for dynamic measurement of stress changes between anchors. The proposed system is also easy in the results interpretation. A change in limit cycle frequency which indicates a change in ceiling stress is easy to find in the frequency spectrum. This is due to the fact the limit cycle frequency has an amplitude of oscillations significantly larger than the amplitude of the disturbance. Hence, in contrast to other measurement systems, it requires less expert knowledge to analyze the results.

The system requires further research work, especially in applying it to the anchoring of less heterogeneous materials. It will be essential to confirm the possibility of interpreting the results for anchoring in multilayer ceilings with different fractions of rock materials.

## Figures and Tables

**Table 1:** The Piezo-accelerometer IMI 623C01 specification.

Sensitivity:	(±5%) 100 mV/g (10.2 mV/(m/s${}^{2}$))
Frequency Range:	(±3 dB) 48 to 900,000 cpm (0.8 to 15,000 Hz)
Sensing Element:	Ceramic
Measurement Range:	±50 g (±490 m/s${}^{2}$)

**Table 2:** Piezoelectric exciter specification.

	Parameter	Piezo: PS-X-03-6/500
1	Weight	40 g
2	Flat frequency range	50 kHz
3	Capacity	<250 nF
4	Stroke	2.4 μm
5	Preload on piezo	400 N
6	Blocking force	5 kN
7	Piezoelectric modulus (d33)	1.22 × 10${}^{-5}$ m/V

**Table 3:** Summary of numerical modeling results for the Vernier excavation.

Type of Bolting	The Hoek–Brown Criterion			
	Mean Stress	Total Strain	Strength Factor	Maximum Axial Force in the Anchorages [MN]
	[MPa]	[m]	[-]	
	[Coverage, [m]]			
Without bolting	4.7/[1.44]	0.014/[0.07]	[1.28]	-
Full-length bolting	4.9/[1.78]	0.012/ [0.11]	[1.21]	0.0179
	**The Coulomb–Mohr Criterion**			
	**Mean Stress**	**Total Strain**	**Strength Factor**	**Maximum Axial Force in the Anchorages [MN]**
	**[MPa]**	**[m]**	**[-]**	
	**[Coverage, [m]]**			
Without bolting	4.7 [9.34]	0.11 [0.43]	9.06	-
Full-length bolting	4.8 [5.41]	0.10 [0.19]	5.49	0.088

**Table 4:** Frequency change of SAS system for main and diagonal directions.

Load Direction	Frequency Difference [Hz] between the Heads Applied in the Direction Consistent with the Load and the Heads on the Other Two Anchors	Frequency Difference [Hz] between Heads Applied in a Different Direction to the Load and Diagonal Heads (Emitter on K5 Anchor)
K1–K2	51.2 ± 0.1	16.7 ± 0.3 (K5–K1/2)
K3–K4	49.2 ± 0.3	15.1 ± 0.7 (K5–K3/4)
K1–K3	42.5 ± 0.6	11.6 ± 0.3 (K5–K1/3)
K2–K4	43.7 ± 0.3	10.2 ± 0.4 (K5–K2/4)

## Data Availability

Not applicable.

## Abbreviations

The following abbreviations are used in this manuscript:

SAS	Self-excited Acoustical System
NDT	Non-Destructive Testing
DOF	Degrees of Freedom
SF	Strength Factor
GSI	Geological Strength Index
FPGA	Field Programmable Gate Array
RTOS	Real Time Operating System
